# The impact of anaphylaxis on the quality of life and mental health of adults

**DOI:** 10.1111/cea.14249

**Published:** 2022-11-06

**Authors:** Rebecca C. Knibb, Aarnoud P. Huissoon, Richard Baretto, Anjali Ekbote, Sham Onyango‐Odera, Cassandra Screti, Kristina L. Newman, Mamidipudi T. Krishna

**Affiliations:** ^1^ School of Psychology, College of Health and Life Sciences Aston University Birmingham UK; ^2^ Department of Allergy and Immunology University Hospitals Birmingham NHS Foundation Trust Birmingham UK; ^3^ Research and Development Heartlands Hospital, University Hospitals Birmingham NHS Foundation Trust Birmingham UK; ^4^ School of Psychology Nottingham Trent University Nottingham UK; ^5^ Institute of Immunology & Immunotherapy University of Birmingham Birmingham UK

**Keywords:** adults, anaphylaxis, mental health, quality of life, stress

**Key messages**

- Poorer anaphylaxis‐specific QoL is significantly related to greater stress, depression, anxiety, demographic and clinical variables.
- Anaphylaxis to food or spontaneous anaphylaxis has the biggest impact on QoL and mental health
- Anxiety, depression, number of reactions and cause should be considered during the management of adult anaphylaxis

To the Editor,

Anaphylaxis is a severe and potentially fatal allergic reaction to food, drugs, general anaesthetic, latex, bee or wasp venom or can occur spontaneously (idiopathic).^1^ The lifetime prevalence of anaphylaxis is approximately 3% in Europe^1^ and there is evidence to suggest that prevalence has increased during the last two decades.^2^ Much of the research investigating the impact of anaphylaxis on people's lives has focused on children and adolescents, showing an association with low quality of life (QoL) and high anxiety.^3^, ^4^, ^5^ Two qualitative studies with adults similarly reported the impact this condition has on QoL and mental well‐being.^6^, ^7^ There are no quantitative studies looking at the psychological impact of anaphylaxis across its variety of causes, on adults. A greater understanding of this might improve the quality of clinical care and help to direct psychological support where necessary. The aim of this study was to assess the impact anaphylaxis has on the QoL and mental health of adults, using validated measures.

This study employed a cross‐sectional survey design. Ethical approval was provided by an NHS Ethics Committee in the United Kingdom (reference: 16/SC/0238). All participants gave written informed consent. Adult participants (aged ≥ 18 years) were recruited from allergy clinics in University Hospitals Birmingham (UHB) NHS Foundation Trust, which receives referrals from Birmingham, Coventry, Warwickshire, Staffordshire, Shropshire and South Wales. All had a diagnosis of anaphylaxis meeting the World Allergy Organization (WAO) diagnostic criteria^8^ as assessed by a specialist in allergy. Participants completed consent and questionnaires in the clinic or took them home for completion, to post back to the study team. Participants completed demographic details and information about their anaphylaxis (data crossed checked with their clinical records), the World Health Organization Quality of Life Scale (Brief version) (*WHOQoL BREF*) to measure generic QoL, the *Anaphylaxis Quality of Life Scale for Adults (A‐QoL‐Adults)*, the *Hospital Anxiety and Depression Scale (HADS)* and the *Perceived Stress Scale (PSS14)*.

A total of 142 adults took part, reporting anaphylaxis mainly to medication, during general anaesthesia, food, bee or wasp venom, latex or had spontaneous anaphylaxis. Sensitivity analysis showed that the study was able to detect medium effect sizes with 80% power, with alpha set at 0.05. Demographic and clinical information is summarized in Table 1, means and standard deviations for all scales are reported in Table 2. Reported stress in this sample of adults with anaphylaxis was significantly higher than the norm value, *t* = 4.98(135), *p* < .001. A total of 23.2% of males (*n* = 13) and 49.4% of females (*n* = 42) reported moderate to severe anxiety. Mean anxiety levels for females were significantly higher than UK norm values, *t* = 3.27(83), *p* = .002, but were not for males (mean = 4.87, SD = 4.72). A total of 16.1% of males (*n* = 9) and 12.4% of females (*n* = 19) reported moderate to severe depression. Mean depression levels for females were significantly higher than UK norm values *t* = 2.93(83), *p* = .004, but were not for males (mean = 3.04, SD = 3.62). Adults with anaphylaxis reported significantly better general physical QoL than UK norms (*t* = −2.10[133], *p* = .03), but significantly poorer social (*t* = 3.34[138], *p* < .001) and environmental QoL (*t* = −7.68[136], *p* < .001). There was no significant difference in psychological QoL. (Table 2).

**TABLE 1:** Demographic information and anaphylaxis characteristics

	*N* = 142, *N* (%)
Mean age in years (SD)	44.41 (17.30)
Age range in years	18–78
Gender	
Male	56 (39.4)
Female	85 (59.9)
Prefer not to say	1 (0.7)
Ethnicity	
White	117 (82.4)
Indian/Pakistani	13 (9.2)
African/Caribbean	4 (2.8)
Prefer not to say	3 (2.1)
Other	5 (3.5)
Highest level of education	
Vocational qualification	20 (14.1)
Secondary/high school level	28 (19.7)
A level/post‐high school level	30 (21.1)
University degree	46 (32.4)
None	8 (5.6)
Mean N of anaphylactic reactions (SD)	3.51 (7.48)
Cause of anaphylaxis	
Food^a^	43 (30.2)
Peri‐operative general anaesthesia	27 (19.0)
Medication/drugs (excluding general anaesthesia)	15 (11.3)
Spontaneous	30 (21.1)
Wasp or bee venom	24 (16.9)
Latex	1 (0.70)
Exercise induced	1 (0.70)
Change in temperature	1 (0.70)
Symptoms	
Difficulty breathing	95 (66.9)
Skin rash	94 (66.2)
Itchy skin	90 (63.4)
Vomiting	30 (21.1)
Swelling of mouth, lips or face	88 (62.0)
Loss of consciousness	24 (16.9)
Drop in blood pressure	64 (45.1)
Under general anaesthetic	21 (14.8)
Brown grading	
Mild/moderate	48 (33.8)
Severe	91 (64.1)
Prescription of an AAI	
Yes	99 (69.7)
How often do you carry your AAI	
Never	8 (5.6)
Rarely	7 (4.9)
Sometimes	9 (6.3)
Most of the time	23 (16.2)
Always	58 (40.8)
Other allergies	
Yes	72 (50.7)
Hayfever	39 (27.46)
Eczema	25 (17.61)
Asthma	41 (28.87)
Family history of allergy	
Yes	42 (29.6)

*Note*: Figures represent mean (SD) or number (%). Where totals do not equal 100% there is missing data; where they total more than 100% participants could select more than one option.

^a^ Food: peanut, tree nut, soya, sesame, lupin, shellfish, fish, eggs and fruit.

**TABLE 2:** Means (and standard deviations) for the WHOQOL BREF, HADS, PSS and A‐QOL‐Adults for all causes of anaphylaxis compared to norm values and across different causes of anaphylaxis

Scale^a^	Food	Venom	Medication	Spontaneous	All causes^b^	Norm value
	*N* = 43	*N* = 24	*N* = 42	*N* = 30	*N* = 142	
A‐QOL‐A						
Total QoL***	2.48 (0.92)	1.71 (0.46)	1.87 (0.92)	2.54 (0.94)	2.18 (0.94)	n.a
Emotional**	2.78 (1.06)	1.89 (0.63)	2.44 (1.17)	2.82 (1.03)	2.54 (1.07)	n.a
Social**	2.03 (0.95)	1.50 (0.48)	1.64 (0.83)	2.21 (1.17)	1.86 (0.94)	n.a
Limitations***	2.70 (1.04)	1.90 (0.76)	1.75 (0.96)	2.50 (1.13)	2.24 (1.07)	n.a
WHOQOL BREF						
Physical**	3.92 (0.85)	4.18 (0.54)	3.41 (0.88)	3.72 (0.88)	15.15 (3.47)*	15.8 (3.8)
Psychological	3.75 (0.77)	3.95 (0.53)	3.57 (0.75)	3.57 (0.70)	14.81 (2.87)	14.7 (3.4)
Social	3.87 (0.93)	4.10 (0.62)	3.73 (1.09)	3.64 (0.94)	15.26 (3.74)***	14.2 (3.5)
Environmental	3.90 (0.69)	4.19 (0.45)	3.91 (0.53)	3.84 (0.80)	15.78 (2.56)***	14.1 (2.3)
HADS						
Anxiety	7.00 (5.18)	4.32 (3.04)	6.75 (4.86)	7.83 (4.98)	6.57 (4.81)	
Women	8.27 (4.85)	4.56 (2.96)	7.00 (4.44)	8.86 (4.83)	7.64 (4.61)**	5.0
Men	4.64 (5.32)	4.15 (3.21)	6.33 (5.63)	5.00 (4.47)	4.87 (4.72)	6.0
Depression*	3.67 (3.95)	1.73 (2.53)	4.63 (4.45)	4.70 (4.36)	3.82 (4.01)	
Women	4.42 (3.90)	1.78 (2.86)	4.96 (4.73)	4.91 (4.64)	4.37 (4.28)**	3.0
Men	2.53 (3.94)	1.69 (2.39)	4.13 (4.08)	4.13 (3.68)	3.04 (3.62)	3.0
PSS						
Stress	23.86 (8.90)	19.43 (6.92)	24.56 (8.71)	23.44 (8.50)	23.24 (8.49)***	19.62 (7.49)

*Note*: Norm values are taken from *Qual Life Res* 2004;13:299–310 for WHOQOL BREF; *J Health Soc Behav* 1983;24:385–396 for PSS14; *Qual Life Res* 2015;24:391–8 for HADS. *p < .05; **p < .01; ***p < .001. Norm data for HADS are median scores; n.a: not available.

^a^ Asterisks in this column relate to significant differences across different causes of anaphylaxis.

^b^ Asterisks in this column relate to significant differences between all causes of anaphylaxis and norm values.

Poorer anaphylaxis‐specific QoL (as measured by the A‐QoL‐Adults) significantly related to greater anxiety (*r* = .69), depression (*r* = .54), stress (*r* = .38) and poorer generic physical (*r* = −.50), psychological (*r* = −.45), social (*r* = −.36) and environmental QoL (*r* = −.48). Those of a younger age (*r* = −.24), those who had experienced a greater number of anaphylactic reactions (*r* = .22) and those who carried their AAI more frequently (*r* = .30), reported significantly poorer anaphylaxis specific QoL. Females reported significantly poorer anaphylaxis specific QoL (mean = 2.50, SD = 0.93) compared to males (mean = 1.73, SD = 0.75), *t* = −5.09(123.01), *p* < .001. There were no significant differences in ethnicity (Table 2).

There were significant differences for depression across the different causes of anaphylaxis, *F*(3,134) = 3.04, *p* < .05. Post hoc tests showed that those with anaphylaxis to medication reported significantly greater depression than those reacting to bee or wasp venom (*p* < .01). There were significant differences across different causes for general physical QoL as measured by the WHOQoL BREF, *F*(3,128) = 4.40, *p* < .01, but not for social, psychological or environmental QoL. Post hoc tests showed that those with anaphylaxis to medication reported significantly worse physical QoL than those reporting anaphylaxis to venom (*p* < .01) (Table 2).

For anaphylaxis‐specific QoL, there were significant differences across the different causes of anaphylaxis, *F*(3,124) = 6.50, *p* < .001. Post hoc tests showed that those with anaphylaxis to food had significantly poorer QoL than those reacting to bee or wasp venom (*p* < .02) or medication (*p* < .05). Similarly, those with spontaneous anaphylaxis reported significantly poorer QoL than those with anaphylaxis to venom (*p* < .01) or medication (*p* < .05). For the sub‐domains of the A‐QoL‐Adults scale, there were significant differences across the different causes for limitations on life, *F*(3,129) = 7.34, *p* < .001, social QoL, *F*(3,130) = 3.83, *p* < .01 and emotional QoL, *F*(3,131) = 4.49, *p* < .01. Post hoc tests showed that those with spontaneous anaphylaxis reported poorer social (*p* < .05) and emotional QoL (*p* = .01) than those reacting to venom. Those reacting to food reported poorer emotional QoL (*p* < .01) than those reacting to venom and greater limitations on life compared to those reacting to venom (*p* < .05) or medication (*p* < .001). Finally, those with spontaneous anaphylaxis reported greater limitations on life than those reacting to medication (*p* < .05) (Table 2).

A hierarchical multiple regression model was run to explore predictors of anaphylaxis‐specific QoL. Demographic and clinical variables were entered in the first step; mental health variables were entered in the second step. The model for the first step (*F*[4,94] = 6.54, *p* < .001) and the second step (*F*[8,94] = 18.55, *p* < .001) were significant, with 60% of variance explained overall. Younger age (β = −.15), a greater number of anaphylactic reactions (β = .24) and greater anxiety (β = .62) were significantly associated with poorer anaphylaxis‐specific QoL, with anxiety the strongest predictor.

This study has shown that anaphylaxis has a significant impact on the QoL and mental health of adults. Adults with anaphylaxis reported greater stress and poorer general QoL in social and environmental domains than norm data. The constant vigilance required to avoid respective triggers, which involves continually assessing risk in their environment, may account for this. Almost half of the female adults in this study reported moderate to severe anxiety levels and both anxiety and depression levels were higher in females compared to norm data. Anxiety due to anaphylaxis has been reported in younger age groups in both males and females.^9^ It is unclear why females in this sample reported particularly high levels compared to males. They may have a greater fear of the consequences of anaphylaxis than males and exploration of beliefs and understanding of anaphylaxis would be useful.

Poorer anaphylaxis‐specific QoL was significantly related to greater stress, anxiety, depression and poorer general QoL across all domains, which highlights the impact anaphylaxis has on the day‐to‐day living of adults with this condition. Gender (with females reporting a greater impact), younger age and number of anaphylactic reactions were also significantly associated with QoL. Clinicians should take particular note of patients with these characteristics, as they may require further support in managing their condition and/or require psychological support to reduce mental distress.

Those with anaphylaxis to food or with spontaneous anaphylaxis reported a bigger impact on their anaphylaxis‐specific QoL compared to those with medication or venom as a cause. This may be due to the level of risk assessment and daily effort needed to avoid allergen/s for those with food allergies, or the extra vigilance for those who do not know what causes their anaphylaxis. Those with anaphylaxis to medication reported greater depression and poorer overall physical QoL in comparison to those with anaphylaxis to venom. Clinicians should therefore be aware of the different aspects of lives that are affected, depending on the cause of anaphylaxis, to ensure appropriate support is in place for optimal allergy management. In regression modelling, younger age, greater number of anaphylactic reactions and greater anxiety were significantly associated with poorer anaphylaxis‐specific QoL. Reducing the number of reactions people experience is therefore key to improving anaphylaxis‐related QoL and it is likely that this may also reduce anxiety, which was the strongest predictor of QoL. Those with high levels of anxiety may benefit from psychological support.

Some limitations should be taken into account when assessing the generalisability of this study. The sample is predominantly White British, almost 60% were educated to at least a post‐high school level (A levels in the UK education system) and data was generated from a single centre, although it is a major regional centre with a wide catchment area. Nevertheless, this study reports novel findings in a sample of well‐characterized adult patients with anaphylaxis which could assist in informing clinical and psychological support for allergy management. Patient education and training for allergen avoidance are needed to improve self‐management of anaphylaxis. In addition, help in recognizing when anaphylaxis is having an impact on mental well‐being, including anxiety and depression, is key to timely referral to psychological support. In particular, those with anaphylaxis to food or spontaneous anaphylaxis may benefit from support to help improve their QoL. Further multi‐centre studies with a demographically more diverse sample may help gain further insight into the impact of anaphylaxis on QoL and facilitate development of novel supportive interventions.

## AUTHOR CONTRIBUTIONS

RK and MTK designed the study protocol; MTK, APH, RB and AE provided access to and helped recruit participants and collect data; SO‐O, CS and KN collected data; RK and CS analysed the data. RK drafted the manuscript which co‐authors reviewed, edited and agreed with the final version.

## FUNDING INFORMATION

Funding for the study was provided by the Department of Allergy and Immunology, University Hospitals Birmingham NHS Foundation Trust.

## CONFLICT OF INTEREST

R Knibb: Work on the project paid for by a grant from the Department of Allergy and Immunology, University Hospitals Birmingham NHS Foundation Trust. RK has received research funding from Aimmune, National Peanut Board, Novartis, NIHR. RK has received the honoraria to give a conference presentation from DBV Technologies. AP Huissoon: Speaker fees ALK Abello. R Baretto: Sponsorship to attend a conference ALK Abello, Novartis. Honoraria for lectures and an educational grant from Thermofisher. A Ekbote: None. S Onyango‐Odera: None. C Screti: Work on the project paid for by a grant from the Department of Allergy and Immunology, University Hospitals Birmingham NHS Foundation Trust. K L Newman: Work on the project paid for by a grant from the Department of Allergy and Immunology, University Hospitals Birmingham NHS Foundation Trust. Mamidipudi T Krishna: Sponsorship from ALK Abello to attend a conference. MTKs department received educational grants from ALK Abello, Thermofisher, MEDA and other pharmaceutical companies for annual PracticAllergy course. MTK has received research funding from NIHR, MRC CiC, FSA and Institute of Global Innovation (University of Birmingham). MTK is chair of Equality, Diversity and Inclusion working group of BSACI.

## Data Availability

The data that support the findings of this study are available on request from the corresponding author. The data are not publicly available due to privacy or ethical restrictions.
